# Hinokitiol for hypertensive emergencies: effects on peripheral resistance, cardiac load, baroreflex sensitivity, and electrolytes balance

**DOI:** 10.1007/s00210-023-02400-0

**Published:** 2023-01-30

**Authors:** Hoda A. Omar, Hany M. El-Bassossy, Noura A. Hassan

**Affiliations:** Department of Pharmacology and Toxicology, Faculty of Pharmacy, Zagazig University, Zagazig, 44519 Egypt

**Keywords:** Hinokitiol, β-Thujaplicin, Hypertensive emergency, Reflex tachycardia, Vasodilators

## Abstract

Hinokitiol, a natural monoterpenoid, has been shown previously to possess a potent vasodilating activity in vitro in both control and hypertensive aortae. Here, the antihypertensive and cardioprotective effects of an intravenous hinokitiol injection were fully investigated in angiotensin II-induced hypertensive emergency in rats. Hinokitiol intravenous injection was prepared in the form of self-nanoemulsifying drug delivery system. Rat’s arterial and ventricular hemodynamics were measured in real-time recordings in addition to surface electrocardiogram while slow injection of cumulative doses of hinokitiol or vehicle as well as time control. Hinokitiol at dose 10 mg/kg showed a considerable reduction in the raised systolic blood pressure (30 mmHg) within only 30 min. The decrease in blood pressure seems to be mediated through a reduction in peripheral resistance, as appears from the decreases in diastolic pressure, dicrotic notch pressure, and pulse pressure. In addition, hinokitiol injection reduced heart load due to the decrease in heart rate, increases in cycle duration (particularly the non-ejection duration) and diastolic duration, and decreases in end-diastolic pressure. An effect most likely mediated via prolongation of ventricular repolarization as appears from the increases in PR, QTc, and JT intervals. However, acute intravenous injection of hinokitiol neither affected the baroreflex sensitivity nor sodium/potassium balance. In conclusion, acute hinokitiol intravenous injection markedly reduced severe hypertension in rats. This effect seems to be mediated through decreasing peripheral resistance and decreasing cardiac load, suggesting that it is an effective treatment in hypertensive emergencies after clinical evaluation.

## Introduction

Hypertension is the leading risk factor for serious diseases such as cardiovascular disease, pre-eclampsia and eclampsia, chronic kidney disease, and a leading cause of premature death worldwide (Petri et al. 2021). Globally, the majority (two-thirds) of the 1.28 billion persons aged 30–79 who have hypertension live in low- and middle-income countries. Undiagnosed hypertension affects 46% of adults. Forty-two percent of hypertensive adults are diagnosed and treated. Twenty-one percent of adults with hypertension have their condition under control (Williams et al. 2018). Hypertensive emergency is defined as acute hypertension-mediated target-organ damage and necessitates prompt and aggressive treatment to prevent further injury. Acute severe hypertension is a common reason for hospitalizations in the USA, accounting for an estimated 4.6% of all visits to emergency departments and hospitalizations (Peixoto 2019).

Due to the severity and prevalence of hypertension, numerous synthetic drugs were developed to treat hypertension. In hypertensive emergencies, direct vasodilators such as sodium nitroprusside, nitroglycerine, fenoldopam, and nicardipine are frequently administered via intravenous infusion (Aronow 2017). While the majority of vasodilators are effective, they can hide their antihypertensive activity by causing reflex tachycardia (Aggarwal and Khan 2006). In addition, some vasodilators pose a number of health risks, including sodium nitroprusside which despite having the lowest purchase cost and the longest track record of efficacy in hypertensive emergencies, is metabolized into lethal thiocyanate and cyanide (Elliott 2006). Therefore, scientific studies recommend using appropriate phytomedicine and lifestyle modification as an alternative method of hypertension management (Jalalyazdi et al. 2019).

Monoterpenes have a great range of pharmacological properties, such as anti-hypertensive, bradycardic, antiarrhythmic, and hypotensive actions. Consequently, they have attracted great interest from the pharmaceutical industry to develop new medications (Silva et al. 2021). In previous studies, several monoterpenes like menthol, thymol, and linalool have been shown to modulate the function of several types of ion channels in different excitable cells like neurons, different smooth muscles, and cardiomyocytes (Oz et al. 2015). The monoterpene of interest in the present study is hinokitiol (β-thujaplicin), which is naturally found in the woody portion of Cupressaceae family trees (Hoang and Han 2020). Recently, we have revealed that hinokitiol can induce vasodilation in aorta isolated from angiotensin II (Ang II)-induced hypertensive rats, an effect mediated through blocking calcium mobilization through voltage-dependent calcium channels, stimulating ATP-dependent K^+^ channels, and through nitric oxide signaling pathway (Abo Laban et al. 2022). Based on hinokitiol effect on ion channels and nitric oxide, we proposed that hinokitiol can be beneficial in modulating arterial and cardiac changes in hypertensive states. So, in the current study, we examined the effects of hinokitiol on an acute model of Ang II-induced hypertension in rats using invasive arterial and left ventricular hemodynamic as well as cardiac conductivity monitoring techniques. Additionally, the effects on spontaneous baroreflex sensitivity (BRS) and electrolyte balance were studied.

## Materials and methods

### Animals

For this study, 6–8 weeks old male Wistar rats weighing 250–275 g were obtained from Zagazig University, Zagazig, Egypt. Each 3–4 rats were housed in polypropylene cages with adequate ventilation, 50–60% relative humidity, 22 ± 2 °C temperature, and 12-h day/night cycle. Rats were provided with an unlimited supply of pelleted rodent food and purified water. The requirements of the Zagazig University Ethical Committee for Animal Care were followed in the experimental planning and animal care procedures (Approval number; ZU-IACUC/3/F/197/2019). Animal studies are reported in compliance with the ARRIVE guidelines (Lilley et al. 2020).

### Invasive blood pressure (BP) monitoring

According to the procedure described in our earlier articles, the BP was monitored invasively and in real-time (Abdallah et al. 2021). For induction of anesthesia, the rats received a single intraperitoneal injection of 10 mg/kg xylazine and 100 mg/kg ketamine. The body temperature of the animals was maintained at 37 °C using a rectal probe and automated heating pads. A microtip pressure–volume catheter (PV catheter, SPR-901, Millar Instruments, Houston, TX, USA) was inserted into the right carotid artery through a small incision. This device can monitor arterial pressure continuously. The BP module (Lab Chart professional software v8.0, AD Instruments, Bella Vista, Australia) was used to monitor all hemodynamic parameters in real-time.

### Cardiac hemodynamics recording

The microtip catheter was inserted into the left ventricle while maintaining precise pressure control. The cardiac hemodynamic signals were recorded before and after hinokitiol administration each for 10 min. The BP module was utilized to quantify diastolic duration and end diastolic pressure (EDP).

### Electrocardiogram (ECG) recording

The standard surface ECG was recorded as described in a previous study by our team (El-Bassossy et al. 2018). The ECG module (Lab Chart professional software v8.0, AD Instruments, Bella Vista, Australia) quantitatively evaluates the various ECG components.

### Acute induction of hypertension

The standard dose of Ang II (120 ng·min^−1^·kg^−1^) commonly used in osmotic mini-pumps (El-Bassossy et al. 2018) was infused gradually with a syringe pump through the femoral vein (Advance Infusion system Series 1200, CellPoint Scientific, Gaithersburg, MD) and continued throughout the experiment. This experimental model of hypertension in rats replicates aspects of human hypertension (Lohmeier 2012). Furthermore, Ang II infusion experiments establish a qualitative and quantitative correlation between elevated plasma Ang II concentrations and elevated pressure in arteries (Preston White et al. 1989).

### Hinokitiol intravenous administration

Rats were divided into three groups (*n* = 6): control group, the vehicle group, and intravenous hinokitiol group. After 10 min of catheter stabilization in the left ventricle, Ang II infusion was initiated, and cardiac hemodynamics were recorded for 10 min. The catheter was removed from the heart and positioned in the carotid artery for arterial hemodynamic measurement. After 5 min of stabilization in the carotid, hinokitiol or vehicle was injected into the femoral vein at cumulative doses of 5 and 10 mg·kg^−1^ every 20 min in a volume of 0.4 ml. The intravenous hinokitiol injection was prepared in the form of self-nanoemulsifying drug delivery system according to our previous study (Abdallah et al. 2021). The dose of hinokitiol was chosen based on previous research (Lahlou et al. 2003; Cho et al. 2011). After 20 min of injecting the final dose of hinokitiol or vehicle, the catheter was advanced into the left ventricle under careful pressure control to record cardiac hemodynamic signals for an additional 10 min. Injections of double-distilled water (0.8 ml) were made during time control experiments.

### Baroreflex sensitivity analysis by sequence method and frequency domain analysis

As in the previous study, spontaneous BRS was calculated using the sequence method and frequency domain analysis with the Nevrocard small animal BRS software package for BRS analysis in small animals (Shaltout and Abdel-Rahman 2005). Sequence method BRS is based on quantifying at least three beats in which systolic arterial pressure (SAP) sequentially increases or decreases along with changes in the R-R interval (RRI) of the subsequent beat in the same direction. In frequency domain analysis, the power spectral densities of SAP and RRI oscillations were computed using 512-point fast Fourier transform and integrated over the specified frequency range: low frequency (LF) of 0.25–0.75 Hz and high frequency (HF) of 0.75–5.0 Hz. To calculate LF-α and HF-α indices, which reflect the BRS, the square root of the ratio between RRI and SAP powers was used.

### Effects of hinokitiol on serum electrolyte levels

Blood samples from the femoral vein were obtained at the end of the experiment. Using a Spectrum colorimetric test kits (Spectrum-Egyptian Co. For Biotechnology) and spectrophotometer, colorimetric methods were used to analyze the blood’s sodium and potassium levels.

### Drugs and chemicals

Hinokitiol (Sigma-Aldrich, Dorset, UK), Ang II (Sigma-Aldrich, Munich, Germany), ketamine (Sigma pharmaceutical industries, Menoufia, Egypt), and xylazine (Seton®, Laboratories Calier, Barcelona, Spain) were used in this study.

### Data and statistical analysis

The present study’s values are presented as mean ± standard error of estimate mean value. The “*n*” values represent independent animals, not replicates. The sample size and number of animals were determined by a power analysis of previously collected data (El-Bassossy et al. 2018; Abdallah et al. 2021). The research used blinded analysis and randomization to create groups of similar size. In addition, all calculations, from lab-chart analysis to statistics, were carried out using fully automated computer programs. The Prism 5 computer program was used for statistical analysis (Graph Pad, USA). Statistical comparison of the change in arterial hemodynamics, ECG, and BRS values was done using baseline-corrected repeated measures two-way ANOVA followed by the Bonferroni’s post hoc test, while the statistical comparison of the change in cardiac hemodynamics parameters and electrolyte balance was performed using one-way ANOVA, followed by Tukey’s post hoc test. The post hoc analysis was only performed if the overall ANOVA *P* value was statistically significant. *P* < 0.05 was considered statistically significant.

## Results

### Effects of intravenous injection of hinokitiol on elevated systolic BP

**Fig. 1:**
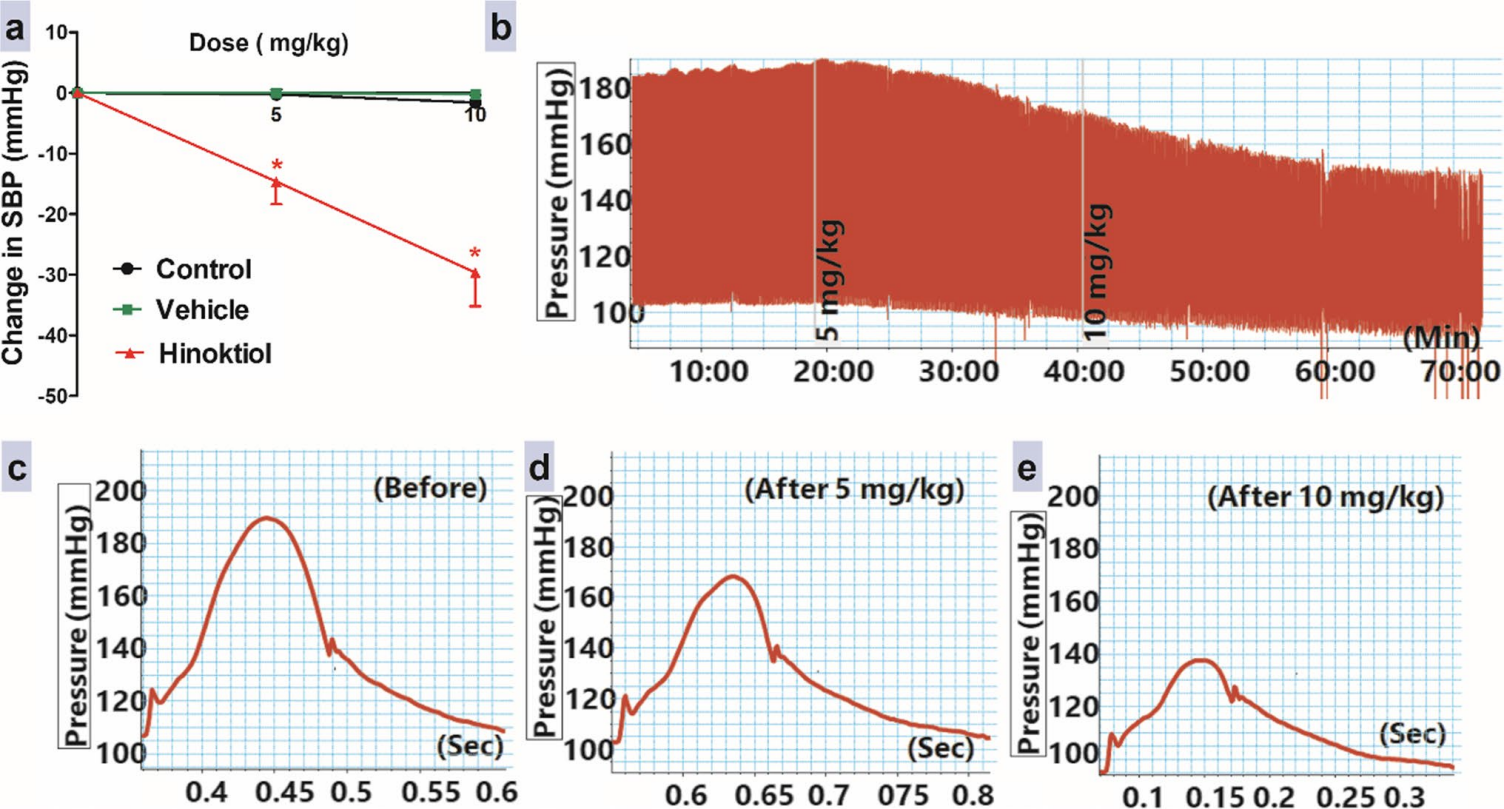
Effects of slow intravenous injection of hinokitiol on the systolic BP (SBP, **a**). Representative original traces of invasive arterial recordings (**b**), before (**c**), and after gradually intravenous injection of hinokitiol in cumulative doses of 5 (**d**) and 10 (**e**) mg·kg^−1^ in rats with an acute model of angiotensin II-induced hypertension. Data are presented as mean ± standard error of six animals. *P < 0.05, in comparison to the respective vehicle values; by two-way ANOVA and Bonferroni post hoc test

In comparison to the vehicle group, intravenous injection of hinokitiol at doses of 5 and 10 mg·kg^−1^ significantly decreased the elevated systolic BP induced by Ang II 20 min after each dose injection (Fig. 1).

### Effects of intravenous injection of hinokitiol on peripheral resistance

**Fig. 2:**
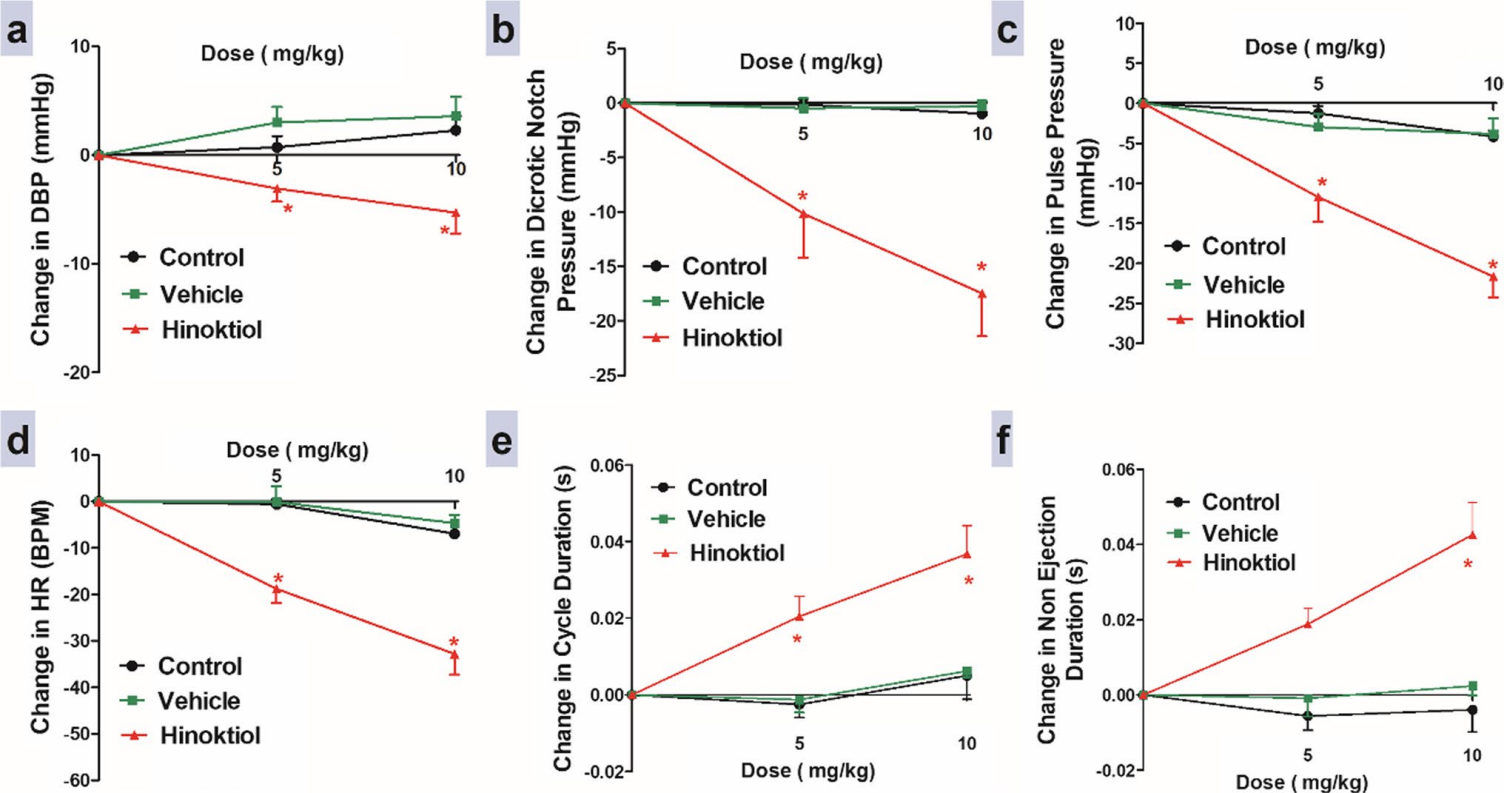
Effects of slow intravenous injection of hinokitiol on the diastolic blood pressure (DBP) (**a**), dicrotic notch pressure (**b**), pulse pressure (**c**), heart rate (HR, **d**), cycle duration (**e**), and non-ejection duration (**f**). Data are presented as mean ± standard error of six animals. *P < 0.05, in comparison to the respective vehicle values; by two-way ANOVA and Bonferroni post hoc test

In comparison to the vehicle group, intravenous injections of 5 and 10 mg·kg^−1^ of hinokitiol resulted in a significant gradual decrease in diastolic BP, dicrotic notch pressure, and pulse pressure (Fig. 2a-c).

### Effects of intravenous injection of hinokitiol on cardiac workload

**Fig. 3:**
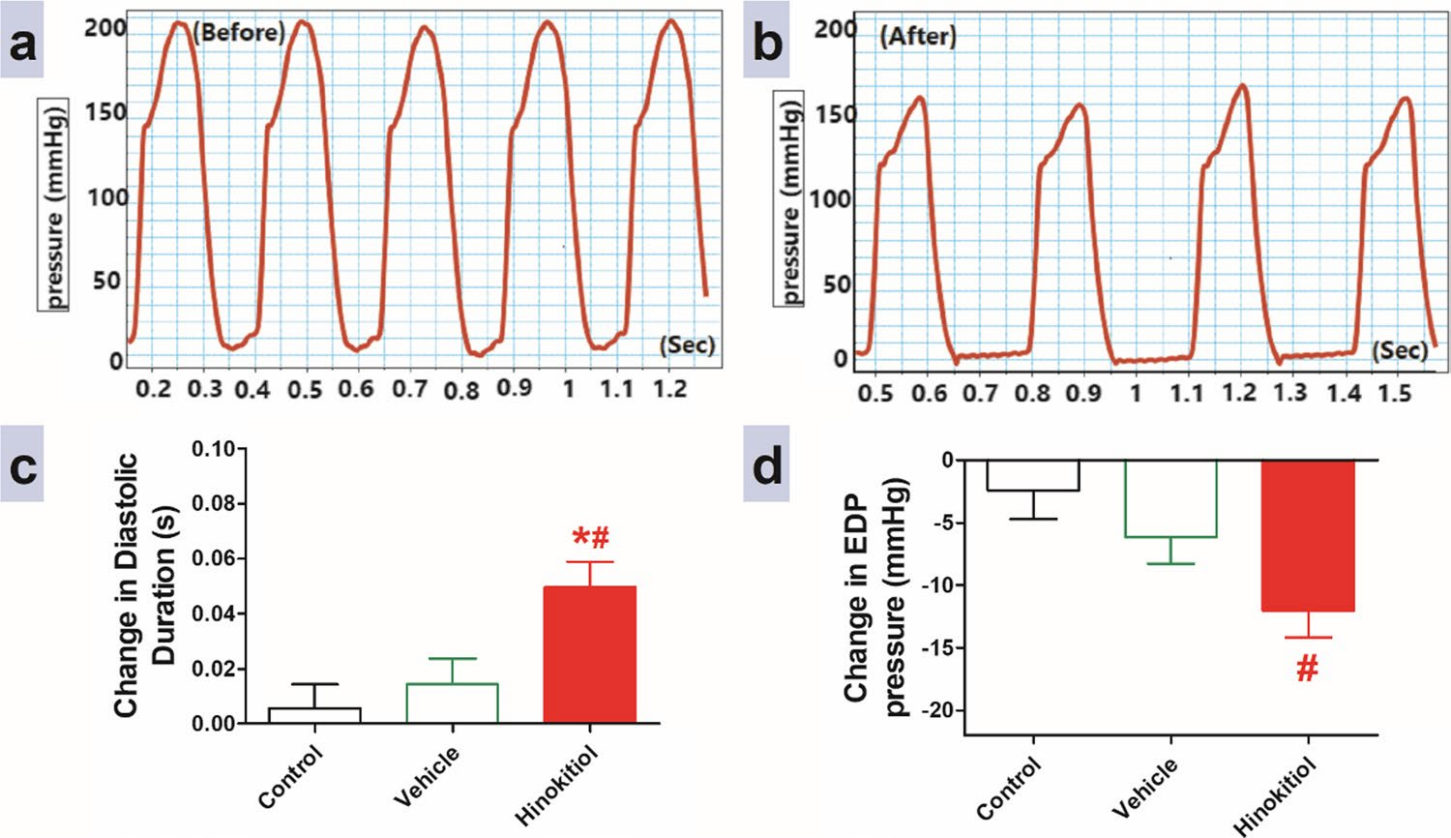
Panels **a** and **b** display representative original recordings of ventricular pressure before and after 10 mg/kg hinokitiol injection for 40 min, respectively. The effects of slow intravenous injection of hinokitiol on diastolic duration (**c**) and end diastolic pressure (EDP, **d**). Data are presented as mean ± standard error of six animals. *P < 0.05, in comparison to the respective vehicle values, #*P* < 0.05, in comparison to the respective time control values; by one-way ANOVA and Tukey’s post hoc test

After 20 min of each dose injection, hinokitiol intravenous injection at doses of 5 and 10 mg·kg^−1^ significantly decreased heart rate (HR) relative to the vehicle group (Fig. 2d). In addition, Fig. 2e and f demonstrate that intravenous injection of hinokitiol resulted in a gradual, dose-dependent increase in cycle duration at doses 5 and 10 mg·kg^−1^ and a significant increase in non-ejection duration at dose 10 mg·kg^−1^ compared to the vehicle group. Moreover, Fig. 3 demonstrates that an intravenous injection of 10 mg·kg^−1^ hinokitiol resulted in a significant increase in diastolic duration compared to the control and vehicle groups. In contrast, intravenous injection of 10 mg·kg^−1^ hinokitiol resulted in a significant decrease in EDP pressure compared to the control group.

### Effects of intravenous injection of hinokitiol on cardiac electrophysiology

**Fig. 4:**
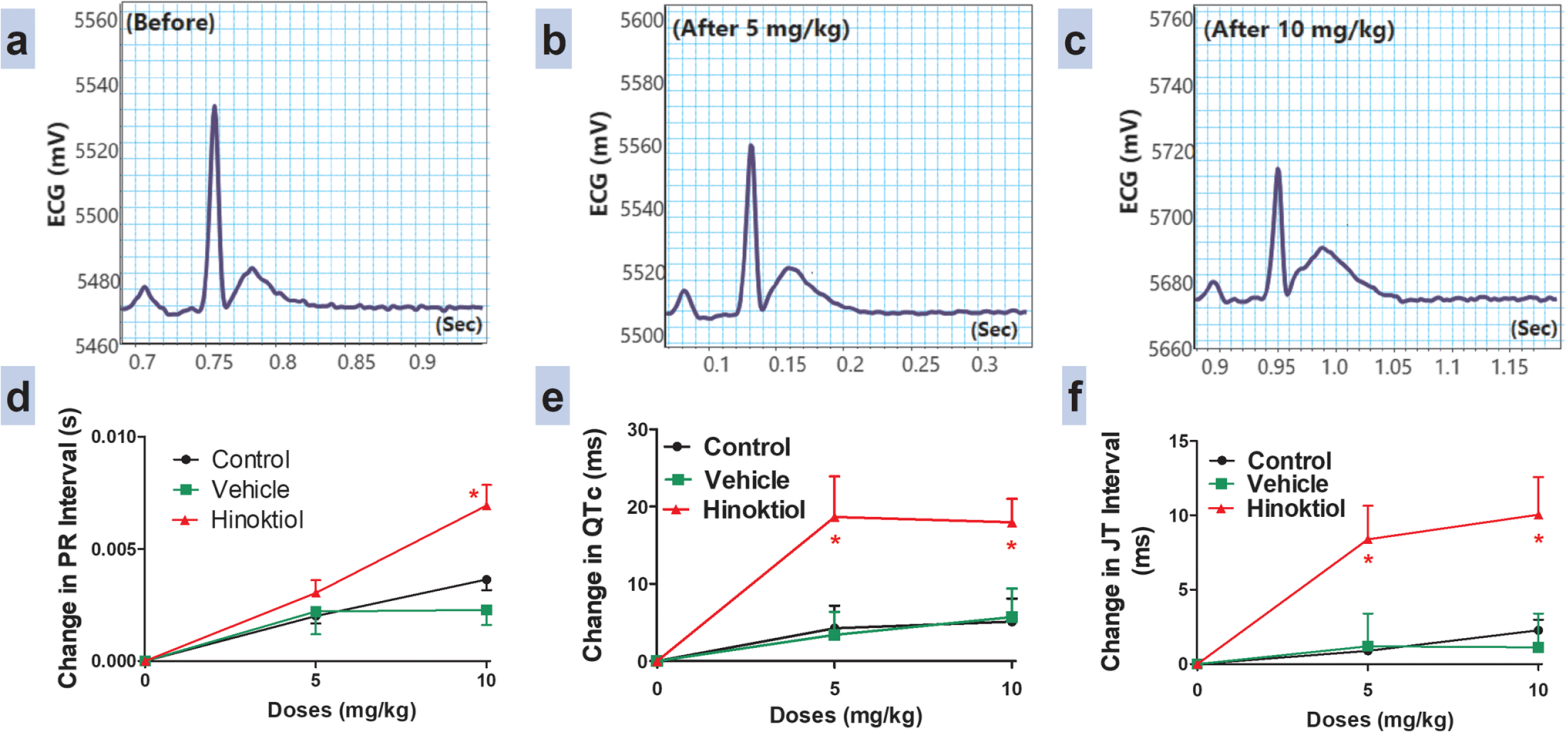
Panels **a**, **b**, and **c** show representative original recordings of ECG before, after 5 mg/kg, and after 10 mg/kg of hinokitiol injection, respectively. Effects of slow intravenous injection of hinokitiol on the PR interval (**d**), QTc (**e**), and JT intervals (**f**). Data are presented as mean ± standard error of six animals. *P < 0.05, in comparison to the respective vehicle values; by two-way ANOVA and Bonferroni post hoc test

In the present study, intravenous injection of hinokitiol at doses of 5 mg·kg^−1^ and 10 mg·kg^−1^ significantly increased PR, QTc, and JT intervals in hypertensive rats infused with Ang II compared to vehicle (Fig. 4a–f).

### Effects of intravenous injection of hinokitiol on baroreceptor sensitivity

**Fig. 5:**
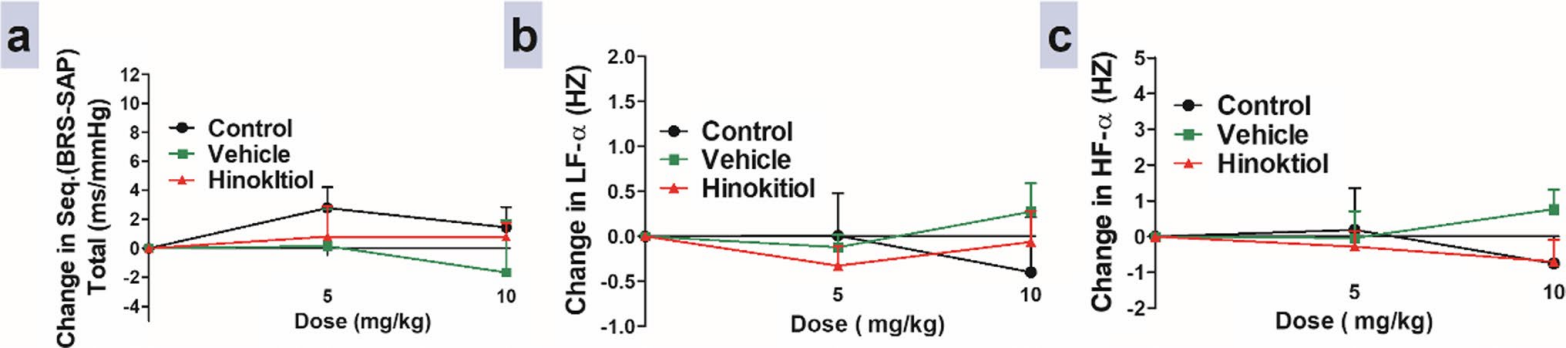
Effects of slow intravenous injection of hinokitiol on the baroreflex sensitivity (BRS) expressed as sequential (BRS: systolic arterial pressure (SAP)) total (**a**), low frequency (LF)-α indices (**b**), and high frequency (HF)-α indices (**c**). Data are presented as mean ± standard error of six animals. *P < 0.05, in comparison to the respective vehicle values; by two-way ANOVA and Bonferroni post hoc test

In the current study, intravenous injection of hinokitiol resulted in non-significant changes in SAP total, LF-α, and HF-α indices in Ang II-infused hypertensive rats when compared to the vehicle group (Fig. 5a–c).

### Effects of intravenous injection of hinokitiol on levels of sodium and potassium in serum

As shown in Table 1, intravenous injection of hinokitiol had no effect on serum sodium or potassium levels when compared to vehicle group.

**Table 1:** Effects of slow intravenous injection of hinokitiol (10 mg/kg) or vehicle on serum sodium and potassium levels after 40 min of injection

Treatment	Serum sodium (mEq/l)	Serum potassium (mmol/l)
Control	104.20 ± 5.40	6.64 ± 0.55
Vehicle	91.68 ± 8.82	6.52 ± 0.49
Hinokitiol	109.40 ± 4.25	6.81 ± 0.42

Data presented as mean ± standard error of six animals. Analysis of data was done by one-way ANOVA and Tukey’s post hoc test.

## Discussion

To the best of our knowledge, this is the first comprehensive investigation of the hypotensive and cardioprotective effects of hinokitiol in a rat model of Ang II-induced hypertensive emergency. Hinokitiol’s effects on arterial and ventricular hemodynamics, ECG, BRS, and serum electrolyte levels were evaluated to determine the drug’s effect on cardiovascular function. Intravenous injection of hinokitiol exhibited a significant antihypertensive effect, as evidenced by a reduction in systolic BP of 30 mmHg in just 30 min, which is compatible with the hypertensive emergency therapy goals that include a 20–25% decrease in mean arterial pressure in the first hour (van den Born et al. 2019). Hinokitiol’s antihypertensive effect is likely mediated by a reduction in peripheral resistance, a decrease in cardiac workload, and modulation of cardiac electrophysiology, without affecting BRS or serum electrolyte levels. The antihypertensive effect of hinokitiol can be explained on the bases of our previous study in which hinokitiol exhibited a significant vasodilating activity in hypertension through nitric oxide dependent mechanism and through calcium channels blocking activity (Abo Laban et al. 2022).

To assess the effect of hinokitiol on peripheral resistance, we examined the effect of hinokitiol on both diastolic BP and dicrotic notch pressure. Clinical data emphasizes that the morphology of the dicrotic notch is related to the value of mean arterial pressure and, therefore, to peripheral vascular resistance (Politi et al. 2016). In the present study, hinokitiol intravenous injection significantly decreased diastolic BP and dicrotic notch pressure, which reflects the ability of hinokitiol to reduce systemic vascular resistance; one determinant of afterload, and improved arterial compliance, which is related to its previously reported vasodilating activity (Abo Laban et al. 2022). Moreover, hinokitiol intravenous injection significantly decreased pulse pressure, which depends on both arterial stiffness and stroke volume, consequently, physicians have attempted for decades to use it to quantify cardiac output (McGee 2018).

Higher HR increases cardiac workload and oxygen demand and reduces coronary perfusion by decreasing diastolic time (Tanna et al. 2019). Furthermore, numerous studies have reported an independent association between HR and cardiovascular morbidity and mortality in hypertensive patients, where HR and BP act in concert to produce cardiovascular complications (Dalal et al. 2019; Cierpka-Kmieć and Hering 2020). Moreover, the majority of vasodilators used in intensive care units, including nicardipine, clevidipine, nitroglycerin, and hydralazine, cause reflex tachycardia, which can mask their antihypertensive activity (Elliott 2006; Peixoto 2019) and may propagate aortic dissection (Estrera et al. 2006). Intriguingly, our research revealed that hinokitiol intravenous injection lowered BP without causing reflex tachycardia, thereby reducing cardiac workload. Furthermore, injecting hinokitiol intravenously into hypertensive rats increased cycle duration, non-ejection duration, and the duration of diastolic phase, which all related to the negative chronotropic action of hinokitiol. The negative chronotropic effect of hinokitiol can be attributed to its ability to block calcium mobilization through voltage gated calcium channels previously mentioned in our study (Abo Laban et al. 2022). Moreover, we also evaluated hinokitiol effects on preload through assessing left ventricular EDP, which was significantly reduced after hinokitiol intravenous injection, reflecting a reduction in cardiac workload. Our results demonstrate a novel medication that can be used to treat hypertensive emergencies without the problems of the conventional vasodilators.

To further explain our results, we studied the changes in ECG signals during hinokitiol intravenous injection. It is commonly known that there is inverse relationship between HR and PR interval (Soliman and Rautaharju 2012). In the present study, hinokitiol intravenous injection resulted in prolongation of PR interval after dose 10 mg·kg^−1^ which indicate a delay in atrioventricular conduction, thus reducing HR. Moreover, hinokitiol intravenous injection led to a significant increase in QTc and JT intervals, which reflects prolonged ventricular repolarization (Rabkin et al. 2017).

The BRS contributes significantly to the neuronal regulation of the cardiovascular system. Moreover, hypertension is linked to a decrease in BRS and autonomic dysfunction (Queiroz et al. 2013). By examining the BRS analysis, we found that acute injection of hinokitiol had no effect on sequential (BRS-SAP) total which reflects the total BRS sensitivity through determining the relationship between the change in R-R interval and systolic BP (Hesse et al. 2007). Also, hinokitiol intravenous injection did not produce change in spectral LF- and HF-BRS responses, which reflect the effects on sympathetic and parasympathetic stimulation, respectively (Alipov et al. 2005). Our results revealed that despite BP lowering activity of hinokitiol and its previously mentioned vasodilating action, it did not produce reflex tachycardia which is known to be mediated through baroreceptor stimulation. Thus, it is obvious that its negative chronotropic action can be through its direct cardio-depressant action which can be attributed to its blocking activity on calcium mobilization through voltage gated calcium channels previously reported (Abo Laban et al. 2022). However, we cannot exclude the possibility of existence of central effects of hinokitiol on HR which is out of the scope of the present study.

To test whether the change in electrolyte levels contribute to the acute antihypertensive effect of hinokitiol, we measured serum sodium and potassium levels. Acute intravenous injection of hinokitiol produced no change in serum sodium or potassium levels, indicating that the acute change in BP is not related to alteration in serum electrolyte levels.

It is obvious from our results that hinokitiol possess an important advantage over the commonly used agents for a hypertensive emergency which is its ability to reduce BP without producing reflex tachycardia; a side effect commonly caused by vasodilators which can worsen ischemia, precipitate angina, and render the drug contraindicated in cases such as aortic dissection (Peixoto 2019). However, to prioritize between hinokitiol and drugs already used to control hypertensive emergencies, more studies must be conducted to exclude the side effects commonly associated with the use of those drugs such as increased intraocular pressure associated with fenoldopam use (Brath et al. 2000), thiocyanate toxicity and increased intracranial pressure associated with nitroprusside use, and asthma associated with the use of beta-blockers (Peixoto 2019).

## Conclusion

In conclusion, vasodilators induce reflex tachycardia and a rise in HR, which can hide their antihypertensive impact and elevates the risk of cardiovascular disease. The efficacy of hinokitiol to diminish the increased systolic pressure, pulse pressure, and dicrotic notch pressure was indicative of its antihypertensive and cardioprotective properties. In addition, the antihypertensive impact of hinokitiol can be mediated via lowering cardiac strain via modification of ventricular diastolic function and electrical activity, without affecting the BRS and sodium/potassium levels. After clinical review, the current study proposes hinokitiol intravenous injection as an effective treatment for hypertensive emergencies.

## Data Availability

The datasets generated during and/or analyzed during the current study are available from the corresponding author on reasonable request.

## Abbreviations

Ang II: Angiotensin II
BP: Blood pressure
BRS: Baroreflex sensitivity
EDP: End-diastolic pressure
HF: High frequency
HR: Heart rate
LF: Low frequency
RRI: R-R interval
SAP: Systolic arterial pressure
